# Glucocorticoid Withdrawal Symptoms and Quality of Life in Patients with Systemic Lupus Erythematosus

**DOI:** 10.1155/2023/5750791

**Published:** 2023-11-10

**Authors:** Matee Karoonkatima, Pongthorn Narongroeknawin, Sumapa Chaiamnuay, Paijit Asavatanabodee, Rattapol Pakchotanon

**Affiliations:** Rheumatic Disease Unit, Department of Internal Medicine, Phramongkutklao Hospital and College of Medicine, Bangkok, Thailand

## Abstract

**Methods:**

SLE patients whose prednisolone had been previously withdrawn or taken <5 mg/day were enrolled. Serum morning cortisol levels were collected after 72-hour GCS discontinuation, and low-dose ACTH stimulation test (LDST) was performed. Patient report outcomes (PROs) included SLE-specific quality of life questionnaire (SLEQoL), functional assessment of chronic illness therapy (FACIT), patient health questionnaire (PHQ-9), and Pittsburgh's sleep quality index (PSQI).

**Results:**

Serum morning cortisol of 100 SLE patients was tested. Most patients were female (88%). Seventy-four patients showed remission. The mean ± SD of prednisolone was 0.73 ± 1.08 mg/day. Total SLEQoL and FACIT (mean ± SD) of all patients were 67.05 ± 26.15 and 13.7 ± 8.87, respectively. Eighteen percent of patients had moderate-severe depressive symptoms, and 49% were poor sleepers. Adrenal function was determined by LDST in only 39 patients; 5 patients (12.8%) were adrenal insufficiency (AI), and 34 patients were normal adrenal function. Compared to normal adrenal function patients, SLE patients with AI had higher proportion of moderate-severe depressive symptom (PHQ − 9 > 9), but not statistically significant (40% vs. 20.6%, *p* = 0.34). PROs were comparable between groups. Independent factors associated with SLEQoL were FACIT (adjusted *β* 1.31, 95% CI 0.76, 1.86, *p* < 0.001), PHQ-9 (adjusted *β* 5.21, 95% CI 4.32, 6.09, *p* < 0.001), and PSQI (adjusted *β* 4.23, 95% CI 3.01, 5.45, *p* < 0.001), but not with AI (adjusted *β* -5.2, 95% CI -33.26, 22.93, 0.71, *p* = 0.71).

**Conclusion:**

SLE patients with previous GCS exposure could experience AI and withdrawal symptoms such as sleep disturbance and depression during discontinuation of low-dose GCS. Fatigue, depression, and poor sleeper were significantly associated with poor SLEQoL.

## 1. Introduction

Systemic lupus erythematosus (SLE) is a complex autoimmune disease, in which inflammatory process is responsible for the main pathogenesis of the disease. Glucocorticoids (GCS) are mainstay treatment of SLE as anti-inflammatory agents. However, long-term use and high dosage of GCS showed increased risk of adverse effects, such as organ damage, infection, osteoporosis, osteonecrosis, Cushing's syndrome, and other systemic diseases [1, 2].

The treat-to-target (T2T) recommendations suggest that treatment in SLE should aim at remission or low disease activity. At these disease states, GCS should be withdrawn or maintained with the lowest possible dose [3]. However, GCS withdrawal in SLE might increase risk of disease flares or contribute to symptoms of GCS withdrawal ranging from mild symptoms such as asthenia, arthralgia, somnolence, and depression to severe adrenal crisis due to adrenal insufficiency (AI). A recent systematic review found that the estimated prevalence of AI among patients with GCS exposure was 37.4% [4]. Moreover, overall health-related quality of life (HRQoL) was reduced among adults with various adrenal diseases [5]. Therefore, we aimed to investigate the GCS withdrawal symptoms and AI and their effects on HRQoL among SLE patients whose prednisolone (PDN) had been withdrawn or tapered less than 5 mg per day.

## 2. Materials and Methods

### 2.1. Study Design and Population

A cross-sectional study was conducted at Rheumatic Disease Unit, Department of Medicine, Phramongkutklao Hospital. Patients were enrolled if they met the 2012 Systemic Lupus International Collaborating Clinic (SLICC) classification SLE criteria and had been followed up in outpatient clinic for at least 6 months [6]. Patients with SLE who had been on PDN ≤ 5 mg/day or off PDN were included. Patients diagnosed with overlapping connective tissue diseases, fibromyalgia, liver cirrhosis, active malignancy, sepsis, and pregnancy or those who took intramuscular or intravenous corticosteroids within 4 weeks were excluded from the study. Ethical review and approval were obtained from the Institutional Review Board of the Royal Thai Army Medical Department (R160h/62). All the participants with the inclusion criteria accepted to enroll in the study after an informed consent had been made.

### 2.2. Clinical Assessment

Clinical data at baseline were collected. Demographic data included gender, age at diagnosis and enrollment, disease duration, comorbidities, and cumulative clinical manifestations. Treatment variables included GCS, antimalarial, and immunosuppressive drugs. Cumulative dosage of GCS and adjusted mean GCS over the 6 months prior enrollment were calculated. Disease activity was assessed by the Systemic Lupus Erythematosus Disease Activity Index 2000 (SLEDAI-2K) [7], and damage was measured by Systemic Lupus International Collaborating Clinic/American College of Rheumatology Damage Index (SDI) [8]. Adjusted mean SLEDAI-2K (AMS), a modified measure adapted from Ibanez et al. [9], was calculated by the summation of SLEDAI-2K over time and then dividing by the length of time for each patient in the past 6 months. AMS represents an average disease activity measure over time.

### 2.3. Hypothalamic-Pituitary-Adrenal Axis (HPA Axis) Test

Patients were screened by unstimulated serum cortisol level during 7 : 00-9 : 00 am. The patients were advised to temporarily discontinue PDN at least 72 hours prior to blood collection. Since discontinuation could exacerbate disease flare up, patients were allowed to resume PDN, if they had any suspicious symptoms. Serum cortisol level and HPA axis assessment was postponed for those patients. The quantitative determination of serum cortisol level was measured by commercial electrochemiluminescence immunoassay (ECLIA) kit. Serum cortisol levels were used to characterize the statuses of relative hypo- or hypercortisolism. The study selected the 25th and 75th percentiles of the unstimulated serum cortisol levels as cut-offs to divide the patients into three groups: the relative hypocortisolism (subjects below the 25th percentile), the relative normocortisolism (subjects between 25th and 75th percentiles), and the relative hypercortisolism (subjects above the 75th percentile). Moreover, AI was defined as those with unstimulated serum cortisol level less than 3 *μ*g/dL or abnormal adrenal function established by low-dose adrenocorticotropic hormone (ACTH) stimulation test (LDST), with a serum cortisol level < 18 *μ*g/dL at 30 or 60 minutes after 1 *μ*g cosyntropin intravenous injection [10].

### 2.4. SLE-Specific Quality of Life Questionnaire (SLEQoL) Assessment

A validated Thai version of the SLEQoL with a recall period of one week was used to measure health-related quality of life (HRQoL) at enrollment. The scores range from 40 to 280, where higher value demonstrates worse quality of life [11].

### 2.5. Functional Assessment of Chronic Illness Therapy (FACIT) Fatigue Scale Assessment

A Thai version of FACIT fatigue scale (with permission from FACIT.org) with a recall period of one week was used to measure fatigue status at enrollment. The scores range from 0 to 52 where higher value demonstrates worse fatigue [12].

### 2.6. Patient Health Questionnaire-9 (PHQ-9) Assessment

A validated Thai version of the PHQ-9 with a recall period of 2 weeks was used as the screening tool for major depression in patients at enrollment. The cut-off score was ≥9 which indicated moderate-severe depressive symptoms [13].

### 2.7. Pittsburgh's Sleep Quality Index (PSQI) Questionnaire Assessment

A validated Thai version of the quality of sleep index using the PSQI with a recall period of 1 month was used to measure the quality and pattern of sleep. The total score ≥ 6 was indicative of a poor sleeper [14].

### 2.8. Statistical Analysis

Descriptive statistics for baseline information and outcomes were presented by mean ± standard deviation (SD), median (interquartile range, IQR), and *n* (%). The difference among groups was compared by one-way analysis of variance (ANOVA). The correlation between serum cortisol level and SLEQoL, FACIT, PHQ-9, and PSQI was calculated by Pearson's correlation. Multivariate regression analyses were performed to identify the independent factors associated with SLEQoL. The statistical software SPSS (version 22) was used for all statistical analyses, and the statistical significance was defined as a *p* value of < 0.05.

## 3. Results

### 3.1. Patient Characteristics

A total of 100 patients with SLE were identified during January 2020 to September 2020. Most patients were female (88%). The mean ± standard deviation (SD) of age at enrollment and disease duration of SLE were 46.89 ± 13.5 and 12.13 ± 9.89 years, respectively. Seventy-four patients were in clinical remission (clinical SLEDAI − 2K = 0). Fifty-nine patients were on PDN. The mean ± SD of the current PDN dose and average dose over the past 6 months were 0.73 ± 1.08 and 1.06 ± 1.81 mg/day, respectively. Blood samples were collected from 100 SLE patients for morning serum cortisol level measurement. Median (IQR) serum cortisol at enrollment was 9.86 (1.94-21.8) *μ*g/dL. Moreover, we identified patients with relative hypo-, eu-, and hypercortisolism based on their serum cortisol levels: hypocortisolism: subjects below the 25th percentile, ≤7.25 mcg/dL; eucortisolism: subjects between 25th and 75th percentiles, 7.256-12.45 mcg/dL; and hypercortisolism: subjects above the 75th percentile, >12.45 mcg/dL. Demographic, comorbid, and treatment were comparable between different statuses of relative hypocortisolism, eucortisolism, or hypercortisolism (Table 1). Patients with relatively hypocortisolism had significantly higher adjusted mean cSLEDAI over the past 6 months compared to the others. Adrenal function was examined by ACTH stimulation test in only 39 patients. Of those, 5 patients (12.8%) had AI, and 34 patients had normal adrenal function. AI occurred over the current dose of PDN range 0-2.5 mg/day. The mean ± SD of PDN dose over the past 6 months of patients in AI and non-AI were 4.65 ± 4.79 and 0.79 ± 1.16 mg/day, respectively (*p* < 0.03). In addition, cumulative PDN dosage over 6 months was significantly higher in patients with AI (0.43 ± 0.36 g) compared to non-AI (0.14 ± 0.21 g) (*p* < 0.04). None of the patients with AI experienced or reported symptoms of adrenal crises.

### 3.2. HPA Axis Test and Patient Report Outcomes (PROs)

The mean ± SD of total SLEQoL and FACIT among 100 total patients were 67.05 ± 26.15 and 13.7 ± 8.87, respectively. Eighteen percent of those patients had moderate-severe depressive symptoms (PHQ − 9 > 9), and 49% were poor sleepers (PSQI ≥ 6). Tables 2 and 3 show SLEQoL, FACIT, PHQ-9, and PSQI in the study population based on serum cortisol levels (*n* = 100) and HPA axis test (*n* = 39). SLEQoL, FACIT, PHQ-9, and PSQI scores were comparable between patients with AI, non-AI, and different statuses of cortisolism. Moderate-severe depressive symptoms were found in 40% of patients with AI and 20.6% of non-AI. However, there was no significant difference between groups (*p* = 0.33). Pearson's correlation found a negative relationship between serum cortisol levels and total SLEQoL, FACIT, PHQ-9, and PSQI scores. Nevertheless, no significant relationship existed between these variables. A detailed presentation of these results is given in supplementary tables 1.

### 3.3. Factors Associated with Health-Related Quality of Life in SLE

Multivariate linear regression analyses identified the independent factors associated with SLEQoL (Table 4). FACIT, PHQ-9, and PSQI increased the likelihood of impaired SLEQoL adjusted for age, cSLEDAI-2K, SDI > 0, and treatment with GCS/immunosuppressive agent (adjusted beta 1.31, 95% CI 0.76-1.86, *p* < 0.001; adjusted beta 5.21, 95% CI 4.32-6.09, *p* < 0.001; and adjusted beta 4.23, 95% CI 3.01-5.45, *p* < 0.001, respectively). Nevertheless, adrenal insufficiency or hypocortisolism was not associated with SLEQoL.

## 4. Discussion

The current study showed that SLE patients who are recently exposed to GCS could experience AI and withdrawal symptoms such as sleep disturbance and moderate-severe depressive symptoms during discontinuation or tapering of PDN less than 5 mg/day. In addition, those symptoms significantly affected the quality of life in SLE. However, GCS withdrawal, or alternatively dosage reduction to <5 mg prednisone equivalent per day, is a therapeutic target when remission or low disease activity of SLE has achieved. The study of Tani et al. showed that 77% of SLE patients successfully stopped GCS without flare during a median follow-up of 2 years [15]. Although disease flares after GCS withdrawal are not common in these particular patients, GCS withdrawal symptoms need to be considered during discontinuation.

In SLE patients, the most common etiology of AI is due to exogenous GCS exposure. Currently, very few studies have studied about the prevalence of AI in SLE patients. To our knowledge, this is the first study to investigate the prevalence of AI in SLE. Our study showed that the prevalence of AI was 12.8% among the patients with SLE who were in remission state and were on prednisone at doses < 5 mg/day. Although the results of the present study were difficult to compare with recent data due to differences in disease and GCS regimens, it showed a trend to be smaller compared to previous studies despite the fact that the HPA axis was evaluated by LDST. Abdu et al. suggested that LDST could replace the conventional 250-microgram ACTH stimulation test (SST) since it was more sensitive and specific compared to SST in the detection of mild GCS deficiency [16]. A systematic review by Joseph et al. demonstrated the percentage of AI in adults with systemic GCS therapy at 37.4% (median 13-63%) [4]. Besides, recent observational studies among patients with systemic inflammatory and autoimmune diseases (lupus nephritis, IgA nephropathy, juvenile idiopathic arthritis, polymyalgia rheumatica, giant cell arteritis, rheumatoid arthritis, and mixed rheumatic diseases) have estimated the prevalence of AI at 15-49% [17–23]. The lower frequency in our population could be mainly explained by 40% of them were off GCS for more than 6 months. On the other hand, the current or over 6-month daily dose of PDN was less than 1 mg/day. This might be the end tail of withdrawal regimen, and HPA axis was recovering as mentioned in previous studies [17, 19, 21]. Another contributing factor was that our patients were suggested >72 hours of withdrawal of GCS prior to HPA axis testing. Henzen et al. observed that the longer duration (>4 days) of GCS discontinuation prior to LDST showed a higher percentage of normal adrenal response [24]. In respect of the factors for HPA axis suppression in patients given GCS therapy, we found that daily and cumulative doses of GCS were significantly higher in patients with AI.

Several GCS withdrawal and adrenal insufficiency symptoms affect daily life's function. Previous studies revealed that AI influenced sleep hygiene, emotion disturbance, and increase risk of psychiatric problems [25–27]. A similar observation was noted by a systematic review of Ho and Druce that quality of life was significantly reduced in patients with AI, irrespective of etiology [5]. In contrast to previous studies, we could not show the association between serum cortisol or AI and PROs. Nevertheless, we observed a greater proportion of moderate-severe depressive symptoms in patients with AI compared to others, but this was statistically nonsignificant. Additionally, serum cortisol levels had a nonsignificant trend of negative relationships with total SLEQoL, FACIT, PHQ-9, and PSQI scores. Interestingly, would various GCS replacement doses improve patient's well-being? A randomised controlled trial conducted by Werumeus Buning et al. revealed that patients with secondary AI receiving a higher dose (0.4-0.6 mg/kg body weight/day) of hydrocortisone (HC) reported better HRQoL on various domains as compared to the lower dose of HC (0.2-0.3 mg/kg body weight/day) for 10 weeks [28]. However, a finding from a meta-analysis including 34 studies showed similar HRQoL scores between higher and lower daily dose of HC replacement among patients with AI [29]. According to our previous study on Thai patients with SLE, Poomsalood et al. revealed that there was no effect of PDN dosage (0 vs. ≤5 vs. >5 mg/day) on impaired SLEQoL after adjusting for disease activity [30]. Therefore, to avoid an excess of GCS replacement therapy, a low dose of GCS was sufficient and could minimize AI symptoms and improve HRQoL. Furthermore, the current study showed that fatigue, depression, and sleeping quality scores were independent factors associated with SLEQoL. Those findings were consistent with previous reports [31–36].

The current study has several potential limitations. First, the study had a small number of participants, and ACTH stimulation test could be performed in only 39 patients. Unfortunately, 61% of the total population could not access the HPA axis test during coronavirus disease (COVID-19) pandemic. The current study shows that AI is more frequent (12.8%) among SLE patients with previous exposure to GCS. However, in the light of small sample size, the burden of AI needs to be interpreted with caution. Actual prevalence of AI should be investigated in a larger sample size. Secondly, the result of PROs from this study was not compared with the control participants whose PDN was not discontinued. Thus, it is difficult to conclude whether high prevalence of somnolence and depression relates to tapering of PDN. In addition, we could not explore the cause of AI such as hemorrhage, infarction, adrenal tumor, or empty sella syndrome which were previously described in literature [37]. Since they had received GCS, it was reasonable to assume that such hypocortisolism is possibly related to GCS-induced AI. Despite limitations, the present study is the first to explore the frequency of GCS withdrawal and AI symptoms and their effects on HRQoL in SLE patients whose GCS was tapered less than the physiological level.

## 5. Summary and Conclusion

Although the current study demonstrated the burden of GCS withdrawal symptom and AI during GCS discontinuation, the authors supported tapering PDN < 5 mg/day when remission or low disease activity was achieved to prevent damage accrual. However, to minimize the sickness or life-threatening adrenal crisis, we highlighted an adrenal function evaluation during initiation of 5 mg-PDN withdrawn. Moreover, gradual withdrawal of 5 mg-PDN might reduce the risk of disease relapse and withdrawal symptoms. At present, the decision to withdraw low-dose GCS by the treating physician needs to weigh up the individual risk of GCS withdrawal symptoms, AI, and disease relapse against GCS-related long-term complications.

## Figures and Tables

**Table 1:** Demographics in the study population based on cortisol levels (*n* = 100).

Variables	Hypocortisolism (*n* = 26)	Eucortisolism (*n* = 49)	Hypercortisolism (*n* = 25)	*p* value^§^	*p* value^¶^
Female (*n*, %)	24 (92.3%)	41 (83.7%)	23 (92%)	0.97	0.29
Age at enrollment (mean ± SD, year)	44.73 ± 12.17	46.51 ± 14.15	49.88 ± 13.49	0.16	0.58
SLE duration (median, IQR, year)	11 (6, 19)	7 (3, 21)	9 (4, 19)	0.79	0.23
Clinical manifestation (*n*, %)					
Lupus nephritis	11 (42.3%)	22 (44.9%)	14 (56%)	0.33	0.83
Neuropsychiatric disorder	4 (15.4%)	4 (8.2%)	1 (4%)	0.17	0.34
Vasculitis	3 (11.5%)	4 (8.2%)	1 (4%)	0.32	0.63
Mucocutaneous disorder	21 (80.8%)	36 (73.5%)	20 (80%)	0.95	0.48
Musculoskeletal disorder	15 (57.7%)	32 (65.3%)	14 (56%)	0.90	0.52
Hematologic disorder	14 (53.8%)	26 (53.1%)	12 (48%)	0.68	0.95
Serositis	2 (7.7%)	7 (14.3%)	4 (16%)	0.36	0.40
Comorbidity at enrollment					
Diabetes (*n*, %)	0 (0%)	6 (12.2%)	1 (4%)	0.30	0.06
Cardiovascular disease (*n*, %)	1 (3.8%)	6 (12.2%)	0 (0%)	0.32	0.23
GFR < 60 mL/min/1.73 m^2^ (*n*, %)	1 (3.8%)	2 (4.1%)	0 (0%)	0.32	0.96
Treatment					
Cumulative doses of prednisolone, 6 months (median, IQR, grams)	0.02 (0, 0.45)	0 (0, 0.26)	0 (0, 0.34)	0.34	0.20
Antimalarial (*n*, %)	22 (84.6%)	43 (87.8%)	21 (84%)	0.95	0.70
Immunosuppressive drugs (*n*, %)	15 (57.7%)	25 (51%)	13 (52%)	0.68	0.58
Laboratories					
Morning cortisol level (median, IQR, *μ*g/dL)	5.86 (4.98, 6.53)	9.87 (8.65, 11.2)	14.1 (12.9, 16.3)	<0.001	<0.001
Disease activity					
Adjusted mean cSLEDAI over the past 6 months (median, IQR)	0.42 (0, 1.5)	0 (0, 0)	0 (0, 0)	0.03	0.03
SDI (median, IQR)	0 (0, 1)	0 (0, 1)	0 (0, 1)	0.86	0.40

^§^Comparison between hypocortisolism and hypercortisolism. ^¶^Comparison between hypocortisolism and eucortisolism. CI: confidence interval; SLE: systemic lupus erythematosus; cSLEDAI: SLE disease activity index (clinical domains); SDI: Systemic Lupus International Collaborating Clinics/American College of Rheumatology Damage Index.

**Table 2:** SLEQoL, FACIT, PHQ-9, and PSQI in the study population based on serum cortisol levels (*n* = 100).

Variables	Hypocortisolism (*n* = 26)	Eucortisolism (*n* = 49)	Hypercortisolism (*n* = 25)	*p* value
Total SLEQoL (mean ± SD)	65.46 ± 28.8	70.02 ± 26.8	62.88 ± 21.93	0.51
Physical (mean ± SD)	8.69 ± 3.48	8.96 ± 4.79	7.88 ± 3.46	0.57
Activity (mean ± SD)	15.81 ± 8.87	16.51 ± 8.98	14.32 ± 6.61	0.57
Symptom (mean ± SD)	14.73 ± 7.66	15.47 ± 7.12	13.76 ± 7.37	0.64
Treatment (mean ± SD)	6.35 ± 2.68	5.94 ± 2.48	5.28 ± 2.32	0.31
Mood (mean ± SD)	6.92 ± 3.76	8.27 ± 5.77	7.44 ± 4.75	0.53
Self-image (mean ± SD)	14.62 ± 5.99	14.94 ± 5.67	14.12 ± 5.9	0.85
FACIT (mean ± SD)	14.54 ± 13.33	13.86 ± 7.05	12.52 ± 6.12	0.71
PHQ-9 (mean ± SD)	4.88 ± 4.02	5.59 ± 3.82	4.12 ± 3.7	0.29
PHQ − 9 > 9 (*n*, %)	5 (19.2)	9 (18.4)	4 (16)	0.95
PSQI (mean ± SD)	7.42 ± 4.11	6.8 ± 3.4	6.84 ± 3.64	0.76
PSQI ≥ 6 (*n*, %)	12 (46.2)	27 (55.1)	10 (40)	0.44

SLE: systemic lupus erythematosus; SLEQoL: systemic lupus erythematosus quality of life; FACIT: functional assessment of chronic illness therapy; PHQ-9: patient health questionnaire; PSQI: Pittsburgh's sleep quality index. PHQ − 9 > 9 indicates moderate-severe depressive symptom; PSQI ≥ 6 indicates poor sleeper.

**Table 3:** SLEQoL, FACIT, PHQ-9, and PSQI in the study population based on hypothalamic-pituitary-adrenal axis (HPA axis) test (*n* = 39).

Variables	Adrenal insufficiency (*n* = 5)	Normal adrenal function (*n* = 34)	*p* value
Total SLEQoL (mean ± SD)	63.6 ± 18.27	69.74 ± 24.55	0.77
Physical (mean ± SD)	9.4 ± 5.50	8.29 ± 2.69	0.93
Activity (mean ± SD)	14.6 ± 7.02	15.82 ± 8.22	0.64
Symptom (mean ± SD)	13.4 ± 4.22	15.5 ± 7.87	0.88
Treatment (mean ± SD)	6.4 ± 3.21	5.74 ± 2.06	0.83
Mood (mean ± SD)	7 ± 2.83	7.56 ± 3.93	0.98
Self-image (mean ± SD)	12.8 ± 4.44	15.29 ± 5.62	0.35
FACIT (mean ± SD)	10.4 ± 4.83	13.38 ± 6.07	0.22
PHQ-9 (mean ± SD)	7 ± 2.74	5.47 ± 3.66	0.24
PHQ − 9 > 9 (*n*, %)	2 (40)	7 (20.6)	0.34
PSQI (mean ± SD)	7 ± 4.3	7.32 ± 3.84	0.77
PSQI ≥ 6 (*n*, %)	2 (40)	18 (52.9)	0.59

SLE: systemic lupus erythematosus; SLEQoL: systemic lupus erythematosus quality of life; FACIT: functional assessment of chronic illness therapy; PHQ-9: patient health questionnaire; PSQI: Pittsburgh's sleep quality index. PHQ − 9 > 9 indicates moderate-severe depressive symptom; PSQI ≥ 6 indicates poor sleeper.

**Table 4:** Multivariate linear regression analyses identified the independent factor associated with SLEQoL.

Variables	Adjusted beta (95% CI)	*p* value
Adrenal insufficiency^¶^	-5.2 (-33.26-22.93)	0.71
Hypocortisolism^¶^	0.99 (0.98-1.01)	0.54
FACIT^§^	1.31 (0.76, 1.86)	<0.001
^∗^PHO9^§^	5.21 (4.32, 6.09)	<0.001
^∗^PHQ9 > 9^§^	44.38 (33.73, 55.02)	<0.001
PSQI^§^	4.23 (3.01, 5.45)	<0.001
PSQI ≥ 6^§^	16.09 (5.55, 26.62)	<0.003

^¶^Adjusting for age, SLE duration, GCS dose, cSLEDAI-2K, SDI, used immunosuppressive agent. ^§^Adjusting for cSLEDAI − 2K > 0, SDI > 0, treatment with GCS/immunosuppressive agent. ^∗^PHQ9/PHQ9 > 9 was analysed with SLEQOL except mood domain. SLE: systemic lupus erythematosus; SLEQoL: systemic lupus erythematosus quality of life; FACIT: functional assessment of chronic illness therapy; PHQ-9: patient health questionnaire; PSQI: Pittsburgh's sleep quality index; CI: confidence interval; GCS: glucocorticoids; cSLEDAI-2K: SLE Disease Activity Index 2000 (clinical domain); SDI: Systemic Lupus International Collaborating Clinic/American College of Rheumatology Damage Index. PHQ − 9 > 9 indicates moderate-severe depressive symptom; PSQI ≥ 6 indicates poor sleeper.

## Data Availability

The data used to support the findings of this study are mainly included within the article and supplementary data.

## Supplementary Materials

Supplementary Materials Supplementary table 1: the correlation between serum cortisol levels and outcomes in the study population (*n* = 100).
