# Supplementary material for: Glucocorticoid Withdrawal Symptoms and Quality of Life in Patients with Systemic Lupus Erythematosus
Source: Int J Rheumatol. 2023 Nov 10;2023:5750791. doi: 10.1155/2023/5750791 (PMC10656199; doi:10.1155/2023/5750791)
Supplement: Supplementary Materials — Supplementary table 1: the correlation between serum cortisol levels and outcomes in the study population (n = 100). [file 5750791.f1.docx]

**Supplementary table 1 The correlation between serum cortisol levels and outcomes in the study population (n=100)**

| **Outcomes** | **Serum cortisol levels** | |
| --- | --- | --- |
|  | ***Pearson correlation*** | ***p*-value** |
| **Total SLE QoL** | -0.007 | 0.95 |
| **Physical** | -0.097 | 0.34 |
| **Activity** | -0.036 | 0.73 |
| **Symptom** | -0.059 | 0.56 |
| **Treatment** | -0.156 | 0.12 |
| **Mood** | 0.062 | 0.54 |
| **Self-image** | 0.081 | 0.43 |
| **FACIT** | -0.069 | 0.50 |
| **PHQ-9** | -0.07 | 0.48 |
| **PHQ-9 > 9** | 0.086 | 0.40 |
| **PSQI** | -0.05 | 0.59 |
| **PSQI ≥ 6** | 0.118 | 0.24 |

SLE, systemic lupus erythematosus; SLE QoL, systemic lupus erythematosus quality of life; FACIT, functional assessment of chronic illness therapy; PHQ-9, patient health questionnaire; PSQI, Pittsburgh sleep quality index; PHQ-9 > 9 indicates moderate-severe depressive symptom; PSQI ≥ 6 indicates poor sleeper.
